# Renal tubular damage and worsening renal function in chronic heart failure: Clinical determinants and relation to prognosis (Bio‐SHiFT study)

**DOI:** 10.1002/clc.23359

**Published:** 2020-04-16

**Authors:** Milos Brankovic, K. Martijn Akkerhuis, Ewout J. Hoorn, Nick van Boven, Jan C. van den Berge, Alina Constantinescu, Jasper Brugts, Jan van Ramshorst, Tjeerd Germans, Hans Hillege, Eric Boersma, Victor Umans, Isabella Kardys

**Affiliations:** ^1^ Department of Cardiology Erasmus Medical Center Rotterdam The Netherlands; ^2^ Department of Internal Medicine, Division of Nephrology and Transplantation Erasmus Medical Center Rotterdam The Netherlands; ^3^ Department of Cardiology Noordwest Ziekenhuisgroep Alkmaar The Netherlands; ^4^ Department of Epidemiology University Medical Center Groningen Groningen The Netherlands

**Keywords:** cardiorenal interaction, heart failure, tubular damage biomarkers, tubular injury, worsening renal function

## Abstract

**Background:**

It is uncertain that chronic heart failure (CHF) patients are susceptible to renal tubular damage with that of worsening renal function (WRF) preceding clinical outcomes.

**Hypothesis:**

Changes in tubular damage biomarkers are stronger predictors of subsequent clinical events than changes in creatinine (Cr), and both have different clinical determinants.

**Methods:**

During 2.2 years, we repeatedly simultaneously collected a median of 9 blood and 8 urine samples per patient in 263 CHF patients. We determined the slopes (rates of change) of the biomarker trajectories for plasma (Cr) and urinary tubular damage biomarkers N‐acetyl‐β‐d‐glucosaminidase (NAG), and kidney‐injury‐molecule (KIM)‐1. The degree of tubular injury was ranked according to NAG and KIM‐1 slopes: increase in neither, increase in either, or increase in both; WRF was defined as increasing Cr slope. The composite endpoint comprised HF‐hospitalization, cardiac death, left ventricular assist device placement, and heart transplantation.

**Results:**

Higher baseline NT‐proBNP and lower eGFR predicted more severe tubular damage (adjusted odds ratio, adj. OR [95%CI, 95% confidence interval] per doubling NT‐proBNP: 1.26 [1.07‐1.49]; per 10 mL/min/1.73 m^2^ eGFR decrease 1.16 [1.03‐1.31]). Higher loop diuretic doses, lower aldosterone antagonist doses, and higher eGFR predicted WRF (furosemide per 40 mg increase: 1.32 [1.08‐1.62]; spironolactone per 25 mg decrease: 1.76 [1.07‐2.89]; per 10 mL/min/1.73 m^2^ eGFR increase: 1.40 [1.20‐1.63]). WRF and higher rank of tubular injury individually entailed higher risk of the composite endpoint (adjusted hazard ratios, adj. HR [95%CI]: WRF 1.9 [1.1‐3.4], tubular 8.4 [2.6‐27.9]; when combined risk was highest 15.0 [2.0‐111.0]).

**Conclusion:**

Slopes of tubular damage and WRF biomarkers had different clinical determinants. Both predicted clinical outcome, but this association was stronger for tubular injury. Prognostic effects of both appeared independent and additive.

## 1 INTRODUCTION

Renal dysfunction is the most prevalent comorbidity among patients with chronic heart failure (CHF) and is strongly associated with clinical outcomes such as heart failure (HF) hospitalization and mortality.^1^, ^2^, ^3^, ^4^ Underlying hemodynamic dependence between the heart and the kidneys is widely considered as the main driver of the cardiorenal interaction leading to adverse outcomes.^5^ However, other biochemical, neurohumoral, metabolic, and immunological derangements also occur during the organs' interplay, which has led to the definition of the cardiorenal syndrome (CRS).^6^, ^7^ Because renal dysfunction entails such a poor prognosis in CHF, attention has focused on identifying the signals along the cardiorenal axis that precede adverse outcomes.^8^ However, the mechanisms and the chronology according to which the failing heart damages‐specific renal structures that lead to CRS are poorly understood.^9^

Decreased baseline renal function is clearly important, but worsening renal function (WRF) quantified as creatinine (Cr) increase over time has been shown to be an even more prominent predictor of adverse outcome in CHF.^1^ We have recently confirmed and extended these findings by using frequent repeated renal function assessments in CHF patients.^4^ In the setting of CHF, WRF may be due to several factors. For example, it may be just temporally reduced due to hemodynamic changes, but it may also be due to permanently reduced renal function from nephron loss.

Renal tubular damage is present in CHF patients due to tubulointerstitial injury by renal tissue hypoperfusion or due to a damaged glomerular filtration barrier.^10^, ^11^, ^12^ Studies have shown that higher levels of urinary tubular damage markers N‐acetyl‐β‐d‐glucosaminidase and kidney injury molecule (KIM)‐1 entailed poor prognosis in CHF independently of eGFR.^4^, ^12^ Therefore, markers of renal tubular damage may reflect another pathway for renal alterations in the milieu of the CRS.

To investigate the degree of tubular injury, we used well‐validated urinary markers^13^, ^14^ such as NAG and KIM‐1 qualified as the biomarker for kidney toxicity in preclinical settings by the U.S. Food and Drug Administration and European Medicines Agency.^15^, ^16^ These urinary markers can both detect and quantify the degree of tubular injury providing discrimination of histopathological severity of the tubular damage caused by both ischemic injury and nephrotoxins.^16^, ^17^, ^18^

There is a potential for simultaneous biomarker‐based monitoring of renal function and tubular status to improve the management of CHF patients during their follow‐up.

## 2 MATERIAL AND METHODS

The serial biomarker measurements and new echocardiographic techniques in chronic heart failure patients result in tailored prediction of prognosis (Bio‐SHiFT) is a prospective cohort of stable patients with CHF, conducted in Erasmus Medical Center, Rotterdam, and Noordwest Ziekenhuisgroep, Alkmaar, The Netherlands. Patients were included if aged ≥18 years and if CHF had been diagnosed ≥3 months ago according to the European Society of Cardiology guidelines (for details, see Figure S1).^19^ Patients were ambulatory and stable, that is, they had not been hospitalized for HF in the past 3 months. The study was approved by the medical ethics committees, conducted in accordance with the Declaration of Helsinki, and registered in ClinicalTrials.gov (NCT01851538). Written informed consent was obtained from all patients. This investigation comprised 263 stable patients with CHF, who were enrolled during the first inclusion period (October 2011 to June 2013) and completed their follow‐up in 2015. Since 95% of the study population had heart failure with reduced ejection fraction (HFrEF), in this study, we focused on the HFrEF patients (n = 250).

All patients were evaluated by research physicians, who collected information on HF‐related symptoms, New York Heart Association class (NYHA) class, and performed a physical examination and collected samples. Information on HF etiology, ejection fraction, cardiovascular risk factors, comorbidities, and treatment were retrieved from hospital records. Study follow‐up visits were predefined and scheduled tri‐monthly (±1 month), with a maximum of 10 study follow‐up visits. All patients were also routinely followed at the outpatient clinic by treating physicians who were blinded for biomarker data. Occurrence of rehospitalizations for HF, myocardial infarction (MI), percutaneous coronary intervention (PCI), coronary artery bypass grafting (CABG), arrhythmias, cerebrovascular accident (CVA), cardiac transplantation, left ventricular assist device (LVAD)‐placement, and mortality was recorded in electronic case‐report forms, and associated hospital records and discharge letters were collected. A clinical event committee, blinded for biomarker data, reviewed hospital records and discharge letters, and adjudicated the study endpoints.

The composite endpoint comprised cardiac death, cardiac transplantation, LVAD implantation, and hospitalization for the management of acute or worsened HF, whichever occurred first. Cardiac death was defined as death from MI or other ischemic heart disease (implantable cardioverter defibrillator (ICD)‐10: I20‐I25), death from other heart disease including HF (I30‐I45 and I47‐I52), sudden cardiac death (I46), sudden death undefined (R96), or unwitnessed or ill‐described death (R98, R99). Hospitalization for acute or worsened HF was defined as a hospitalization for an exacerbation of HF symptoms, in combination with two of the following: BNP or NT‐proBNP >3 times the upper limit of normal, signs of worsening HF, such as pulmonary rales, raised jugular venous pressure or peripheral edema, increased dose or intravenous administration of diuretics, or administration of positive inotropic agents.^19^

Blood and urine samples were collected at baseline and during study visits, and were processed and stored at −80°C. Laboratory personnel was blinded for clinical data. Batch analysis of serum was performed at Erasmus Medical Center: NT‐proBNP was analyzed using an electrochemiluminescence immunoassay (Roche Diagnostics, Elecsys 2010, Indianapolis, Indiana), cardiac troponin T was also measured using an electrochemiluminescence immunoassay (Roche Diagnostics, Elecsys 2010 immunoassay analyzer). Plasma and urine samples were transported at −80°C to HaemoScan BV, Groningen, the Netherlands for batch analysis. Creatinine was determined by a colorimetric test by the Jaffé reaction. Plasma was used undiluted, and urine was diluted 10 times in water (lower limit of detection (LLD): plasma 0.14 mg/dL, urine: 1.56 mg/mL). KIM‐1 was determined in urine diluted 50% in 0.1% BSA/PBS buffer, by ELISA (R&D systems, Minneapolis, Minnesota) (LLD: 0.146 ng/mL). NAG was determined using a substrate p‐nitrophenyl N‐acetyl‐β‐d‐glucosaminidase at pH 4.5 (Sigma, St Louis, Missouri) (LLD: 0.485 U/L). All urinary biomarkers were normalized to urinary Cr concentrations to correct for concentration or dilution of urine. The glomerular filtration rate (GFR) was determined by the Chronic Kidney Disease Epidemiology Collaboration equation that has been validated in HF patients^20^ and categorized using K/DOQI guidelines.^21^

To assess patient‐specific slopes (rates of change over time) of biomarker trajectories, we performed joint modeling (JM) of linear mixed‐effects (LME) and Cox regression models.^22^ The LME models estimate the individual biomarker trajectory based on repeated measurements and also correct for biomarkers' sampling variability (for details, see Figure S2).^23^ The JM then combines LME with the Cox regression model to adjust the biomarker trajectory for different follow‐up durations between patients.^23^ The degree of tubular injury was ordered according to the slopes of tubular damage biomarkers: increase in neither, increase in either, and increase in both; WRF was defined as increasing Cr slope.

For continuous variables, the presence of a linear trend across different categories of renal tubular damage and WRF was assessed by analysis of variance or the Kruskal‑Wallis test and categorical variables were tested by the *χ* ^2^ trend test. Covariates that were univariably associated with tubular damage or WRF (exploratory *P* < .10) were entered into a multivariable logistic regression model applying proportional odds ordinal regression or binary logistic regression.

To investigate endpoint‐free rates, we used the two‐sided Breslow test and the Breslow method to estimate event‐time distributions. The Cox regression model was performed to assess hazard ratios (HR) with 95% confidence intervals (95%CI) for study endpoints.

Statistical adjustments were performed by using biomarkers of interest plus age, sex, diabetes, atrial fibrillation, NYHA class, diuretics, systolic blood pressure, estimated glomerular filtration rate (eGFR) (only for tubular damage markers), and biomarkers of myocardial stretch and damage NT‐proBNP and hs‐cTnT.

Data on all variables were complete, except for systolic blood pressure, which was missing in <5% of patients and for which imputations were applied using patients' clinical and outcome data.

All tests were two‐tailed and *P*‐values <.05 were considered statistically significant. All analyses were performed with SPSS (SPSS 25.0; IBM Corp., Armonk, NewYork),^24^ and R^25^ using the package JMbayes.^26^

## 3 RESULTS

Table S1 summarizes the baseline characteristics of the 250 HFrEF patients. During a median of 2.2 (IQR: 1.4‐2.5) years, we collected a median of nine blood 5, 6, 7, 8, 9, 10 and eight urine 5, 6, 7, 8, 9, 10 samples per patient. Table 1 shows that patients with greater tubular damage during follow‐up, had higher baseline NT‐proBNP, cardiac troponin‐T, and Cr levels (ie, lower eGFR); lower left ventricular (LV) ejection fraction, more frequently diabetes, NYHA class III/IV, and cardiac resynchronization therapy (CRT), and were older. After multivariable adjustments, higher baseline NT‐proBNP and lower eGFR remained independent predictors of more severe tubular damage (per doubling of NT‐proBNP adjusted odds ratio, adj. OR, 1.26 [95%CI 1.07‐1.49], *P* = .006; and per 10 mL/min/1.73 m^2^ eGFR decrease 1.16 [1.03‐1.32], *P* = .015) (Table 3).

**Table 1:** Patient characteristics stratified by NAG and KIM‐1 slopes

	NAG and KIM‐1 stable/decreased (n = 66)	NAG or KIM‐1 increased (n = 104)	NAG and KIM‐1 increased (n = 80)	*P*‐value
Clinical features				
Age (years)	65 (57‐72)	68 (60‐77)	70 (60‐80)	.016*
Men	48 (73)	77 (74)	59 (74)	.90
Ischemic etiology	27 (41)	48 (46)	41 (51)	.21
BMI kg/m^2^	27.4 (25.1‐30.9)	26.2 (24.0‐30.0)	26.3 (24.2‐30.2)	.39
Heart rate b.p.m.	66 (60‐74)	66 (59‐71)	69 (60‐76)	.16
SBP mmHg	121 (110‐134)	120 (105‐140)	120 (108‐130)	.69
DBP mmHg	74 (61‐82)	74 (64‐80)	70 (60‐78)	.05
Congestion^a^	37 (56)	68 (65)	52 (65)	.29
NYHA III/IV	9 (14)	23 (22)	30 (38)	.001*
CRT	26 (39)	34 (33)	18 (23)	.027*
Echocardiographic features^b^				
LVEF	31 (26‐40)	30 (23‐35)	28 (20‐35)	.03*
DiasLVD	62 (56‐67)	64 (57‐72)	65 (57‐74)	.06
SysLVD	49 (42‐56)	51 (42‐59)	53 (43‐62)	.07
E/A ratio	0.7 (0.6‐1.1)	1.0 (0.7‐1.4)	0.9 (0.6‐1.9)	.06
E/E′ ratio	9.7 (6.3‐13.0)	10.9 (6.6‐17.4)	11.4 (7.1‐19.2)	.25
Medical history				
Prior MI	22 (33)	39 (38)	34 (43)	.25
Atrial fibrillation	23 (35)	43 (41)	31 (39)	.66
Diabetes	14 (21)	31 (30)	32 (40)	.014*
Hypertension	26 (39)	47 (45)	40 (50)	.20
COPD	8 (12)	10 (10)	13 (16)	.41
Medication prevalence (%)/average total daily dose (mg)^c^				
Beta‐blocker	96%/45 mg	91%/41 mg	84%/47 mg	.30^d^
ACE‐I/ARBs	96%/24 mg	93%/25 mg	94%/24 mg	.96^d^
Loop diuretics	85%/77 mg	90%/78 mg	96%/97 mg	.15^d^
MRAs	74%/23 mg	70%/23 mg	65%/23 mg	.96^d^
Cardiac biomarkers				
NT‐proBNP ng/L	578 (153‐1680)	1076 (378‐2148)	1682 (866‐3529)	<.001*
cTnT ng/L	12.4 (7.5‐24.8)	16.9 (9.4‐32.4)	22.6 (13.7‐43.3)	<.001*
Renal glomerular indices (plasma)				
Creatinine mg/dL	1.10 (0.92‐1.26)	1.17 (0.97‐1.43)	1.33 (1.04‐1.77)	<.001*
eGFR_mL/min/1.73 m_^2^	70 (51‐79)	58 (44‐76)	50 (36‐72)	<.001*
eGFR<60	21 (32)	57 (55)	52 (65)	<.001*
Renal tubular markers (urine)				
NAG U/gCr	5.1 (2.7‐10.0)	5.7 (3.9‐9.1)	6.7 (4.6‐9.2)	.11
KIM‐1 ng/gCr	452 (238‐930)	485 (243‐882)	555 (256‐973)	.45

*Note:* For reasons of uniformity continuous variables are presented as medians (25th‐75th percentiles) and categorical variables are presented as n (%); *P*‐values signify trend across groups and the asterisk indicates *P* < .05.

Abbreviations: ACE‐I, angiotensin‐converting enzyme inhibitors; ARB, angiotensin II receptor blockers; A, peak late filling velocity; BMI, Body mass index; COPD, chronic obstructive pulmonary disease CRP, C‐reactive protein; cTnT, cardiac troponin T; CVA, cerebrovascular accident; DBP, Diastolic blood pressure; DiasLVD, diastolic left ventricular diameter; E, peak early filling velocity; E′, early diastolic mitral annular velocity; eGFR, estimated glomerular filtration rate; KIM‐1, kidney injury molecule‐1; MI, myocardial infarction; MRA, mineralocorticoid receptor antagonist; NAG, N‐acetyl‐β‐D‐glucosaminidase; NYHA class, New York Heart Association class; SBP, Systolic blood pressure; SysLVD, systolic left ventricular diameter; TIA, transitory ischemic attack.

a Congestion was considered present if ≥2 symptoms or signs were present at baseline (dyspnea, orthopnea, fatigue, elevated jugular venous pressure, presence of rales/crackles and pedal oedema).

b Because of logistic reasons, baseline LVEF, DiasLVD, and SysLVD were available in 74%, E/A ratio in 62%, and E/E′ ratio in 69% of all HFrEF patients.

c Table S3 shows the conversion factors for calculation of total daily dose equivalents of different HF medications.

d *P*‐value for the difference in average total daily dose.

Table 2 shows that patients with Cr incline during follow‐up had higher baseline NT‐proBNP, cardiac troponin‐T and higher eGFR, higher systolic LV diameter, and E/E' ratio, more frequently a history of myocardial infarction, and were on higher loop diuretic doses and lower mineralocorticoid receptor antagonist (MRA) doses. After multivariable adjustments, higher eGFR levels and higher loop diuretic doses, and lower MRA doses, remained independent predictors of Cr incline (per 10 mL/min/1.73 m^2^ eGFR decrease: OR 0.73 [95%CI: 0.63‐0.85], *P* < .001; per 40 mg furosemide equivalent dose increase: 1.30 [1.07‐1.59], *P* = .010; and per 25 mg spironolactone equivalent dose decrease: 1.85 [1.10‐3.09], *P* = .019) (Table 3).

**Table 2:** Patient characteristics stratified by creatinine slope

	Creatinine stable/decreased (n = 104)	Creatinine increased (n = 146)	*P*‐value^a^
Clinical features			
Age years	66 (57‐74)	68 (60‐77)	.18
Men	73 (70)	111 (76)	.30
Ischemic etiology	45 (43)	71 (49)	.40
BMI kg/m^2^	26.6 (24.1‐30.2)	26.8 (24.4‐30.2)	.83
Heart rate b.p.m.	68 (59‐77)	65 (60‐72)	.33
SBP mmHg	121 (110‐136)	120 (106‐132)	.26
DBP mmHg	75 (65‐80)	70 (60‐80)	.08
Congestion*	67 (64)	90 (62)	.65
NYHA III/IV	29 (28)	33 (23)	.34
CRT	35 (34)	43 (30)	.48
Echocardiographic features^b^			
LVEF	31 (23‐40)	29 (23‐36)	.20
DiasLVD	64 (56‐71)	64 (59‐72)	.47
SysLVD	47 (41‐58)	52 (45‐60)	.043*
E/A ratio	0.8 (0.6‐1.3)	0.9 (0.6‐1.3)	.20
E/E′ ratio	9.6 (5.8‐13.3)	11.8 (7.9‐19.0)	.010*
Medical history			
Prior MI	32 (31)	63 (43)	.047*
Atrial fibrillation	35 (34)	62 (43)	.16
Diabetes	27 (26)	50 (34)	.16
Hypertension	41 (39)	72 (49)	.12
COPD	9 (9)	22 (15)	.13
Medication prevalence (%)/average total daily dose (mg)			
Beta‐blocker	89%/48 mg	91%/41 mg	.32
ACE‐I/ARBs	96%/23 mg	93%/25 mg	.21
Loop diuretics	89%/63 mg	93%/98 mg	.003*
MRAs	71%/25 mg	69%/21 mg	.022*
Cardiac biomarkers			
NT‐proBNP ng/L	894 (279‐2158)	1369 (514‐2871)	.042*
cTnT ng/L	14.3 (8.3‐29.4)	20.1 (10.7‐38.1)	.018*
Renal glomerular indices (plasma)			
Creatinine mg/dL	1.32 (1.08‐1.67)	1.10 (0.92‐1.38)	<.001*
eGFR_mL/min/1.73 m_^2^	51 (37‐71)	65 (48‐82)	<.001*
eGFR<60	66 (64)	64 (44)	.002*
Renal tubular markers (urine)			
NAG, U/gCr	5.5 (3.4‐8.5)	6.5 (3.9‐9.3)	.06
KIM‐1, ng/gCr	467 (244‐828)	505 (247‐995)	.21

*Note*: For description, please see Table 2; *P*‐values signify a trend across groups and the asterisk indicates *P* < .05.

a *p*‐value for the difference in the average total daily dose.

b Because of logistic reasons, baseline LVEF, DiasLVD, and SysLVD were available in 74%, E/A ratio in 62%, and E/E′ ratio in 69% of all HFrEF patients.

**Table 3:** Independent predictors of renal tubular damage and worsening renal function

	Multivariable model*	
	OR (95%CI)	*P*‐value
Renal tubular damage (dependent variable)^a^		
NT‐proBNP (per doubling)	1.26 (1.07‐1.49)	*P* = .006
eGFR (per 10 mL/min/1.73 m^2^ decrease)	1.16 (1.03‐1.32)	*P* = .015
WRF (dependent variable)^b^		
Loop diuretics (per 40 mg furosemide equivalent. dose increase)	1.30 (1.07‐1.59)	*P* = .010
MRAs (per 25 mg spironolactone equivalent. dose decrease)	1.85 (1.10‐3.09)	*P* = .019
eGFR (per 10 mL/min/1.73 m^2^ decrease)	0.73 (0.63‐0.85)	*P* < .001

*Note:* OR indicates odds ratio for having a more severe tubular damage or WRF; 95%CI indicates 95% confidence interval for the corresponding OR; eGFR indicates estimated glomerular filtration rate, MRAs indicates mineralocorticoid receptor antagonists.

a Covariates that were found to be different across categories of tubular damage with *P* < .10 (see Table 1) were entered into a multivariable ordinal regression model and those were: age, NYHA class, diabetes, use of cardiac resynchronization therapy (CRT), diastolic blood pressure, NT‐proBNP, cTnT, and eGFR. *Represents only covariates with *P*‐value <.05 were presented in the table.

b Covariates that were found to be different between WRF patient and non‐WRF patients with *P* < .10 (see Table 2) were entered into a multivariable binary regression model and those were: diastolic blood pressure, NT‐proBNP, hs‐cTnT, eGFR, urinary NAG, prior myocardial infarction, loop diuretics and MRAs doses.

**Figure 1:**
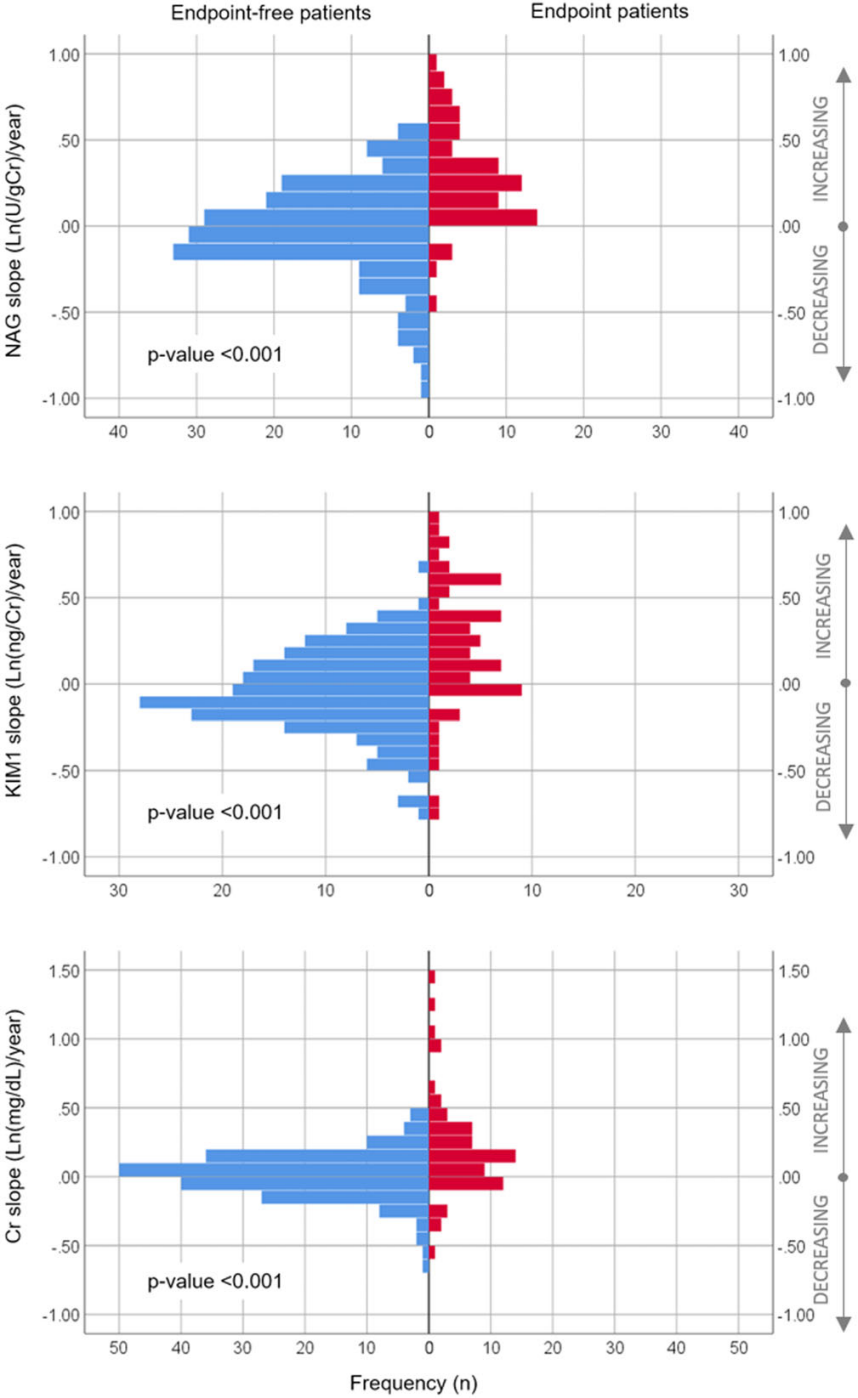
Distributions of slopes of renal biomarkers prior to study endpoints. *Notes:* X‐axis displays number of patients who experienced the event (red) and those who did not (blue), Y‐axis displays the estimated slopes on the continuous scale, where positive numbers correspond to increasing slopes and negative numbers correspond to decreasing slopes. *t* test was used test the average difference between patient with and without event

Of the 250 HFrEF patients, 66 (26%) reached the endpoint: 53 patients were rehospitalized for acute or worsened HF, 8 died of cardiovascular causes, 2 underwent LVAD‐placement, and 3 underwent heart transplantation. Figure 1 shows that patients who experienced the endpoint had significantly higher slopes of urinary NAG than endpoint‐free patients (mean ± SD: 0.27 ± 0.28 vs −0.02 ± 0.27 ln[U/gCr]/year, *P* < .001) and KIM‐1 (0.22 ± 0.36 vs −0.05 ± 0.24 ln[ng/gCr]/year, *P* < .001), and plasma Cr (0.20 ± 0.35 vs 0.01 ± 0.17 ln[mg/dL]/year, *P* < .001). Lower baseline eGFR was positively associated with greater tubular damage but inversely associated with WRF during follow‐up (Table S2).

**Figure 2:**
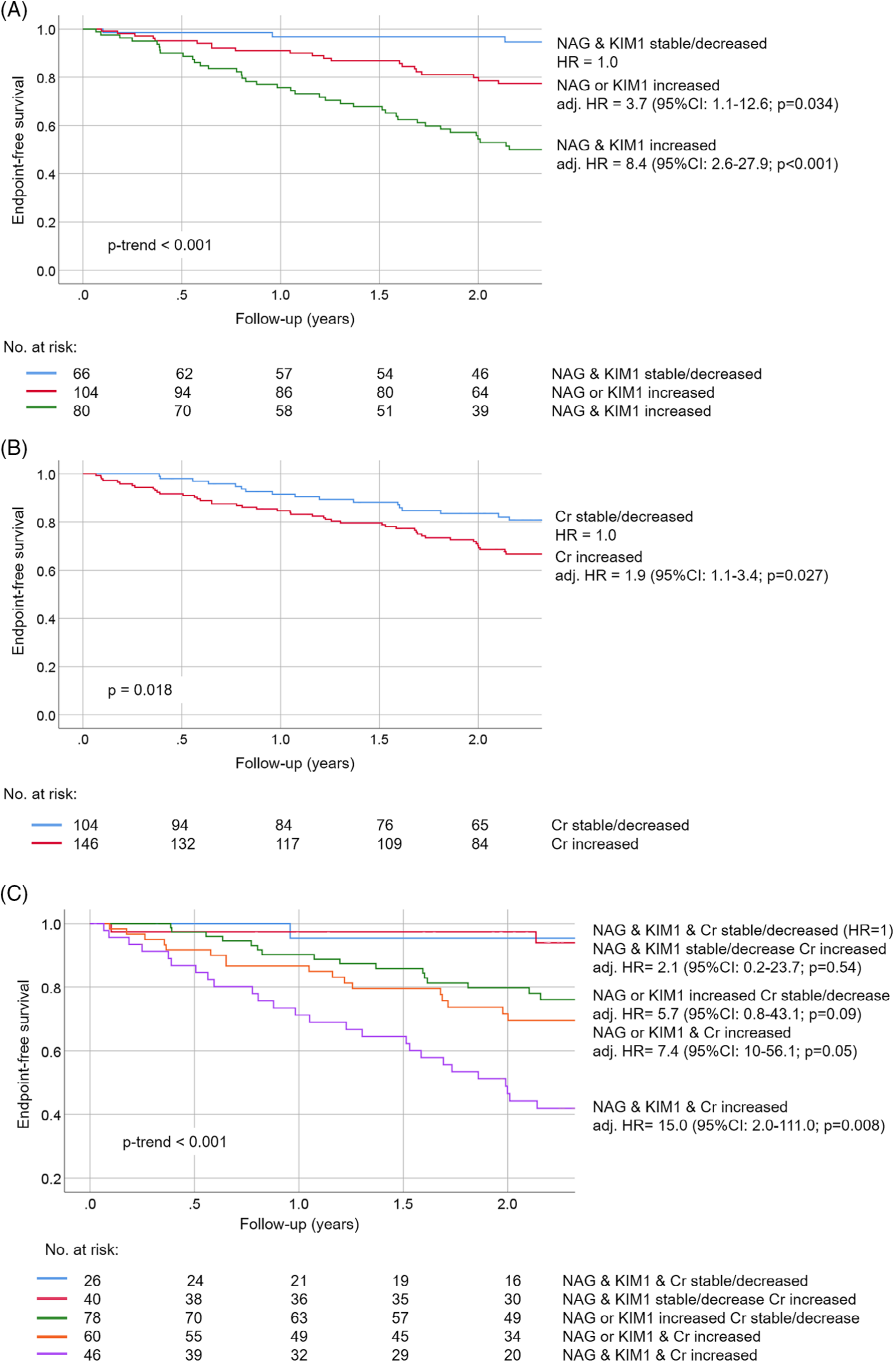
Kaplan–Meier survival curves stratified by slopes of renal biomarkers. *Notes:* Shown are Kaplan–Meier (KM) curves for the cumulative event‐free survival of the composite of HF‐rehospitalization, cardiac death, LVAD placement, and heart transplantation. A, KM curves are stratified by whether both NAG and KIM‐1 slopes were decreasing/stable (blue); either NAG or KIM‐1 slope was increasing (red); or both NAG and KIM‐1 slopes were increasing (green); B, KM curves are stratified by whether Cr slope was decreasing/stable (blue) or increasing (red). C, KM curves are stratified by whether slopes of all three renal biomarkers were decreasing/stable (blue); NAG and KIM‐1 slopes were decreasing/stable, but creatinine slope was increasing (red); either NAG or KIM‐1 slope was increasing but creatinine slope was decreasing/stable (green); either NAG or KIM‐1 slope was increasing, and creatinine slope was increasing (orange); and slopes of all three biomarkers were increasing (purple). Hazard ratios (HR) were adjusted for age, sex, diabetes, atrial fibrillation, NYHA class, diuretics, systolic blood pressure, eGFR (only for tubular damage biomarkers), NT‐proBNP, and hs‐cTnT

Seventy‐four percentage of HFrEF patients experienced incline in either tubular damage biomarker during follow‐up. Of those, 44% of patients had both tubular biomarkers rising prior to the endpoint or last sampling moment. Figure 2A shows that endpoint‐free rates were lowest when both tubular damage biomarkers were increased, followed by the rates when either marker was increasing (*P* for trend <.001). HR were almost four times higher in patients in whom either tubular damage marker was increasing and eight times higher if both were increasing during follow‐up (NAG or KIM‐1 slope increased: adjusted hazard ratios, adj. HR 3.7 [95%CI: 1.1‐12.6], *P* = .034; NAG and KIM‐1 slopes increased: 8.4 [2.6‐27.9], *P* < .001). These estimates were independent of the patients' clinical characteristics, baseline eGFR, and cardiac biomarkers (NT‐proBNP and troponin T).

Fifty‐eight percent of HFrEF patients experienced incline in Cr levels during follow‐up. Figure 2B shows that patients with increasing plasma Cr slope had lower endpoint‐free rates than their counterparts (*P* = .018). The HR in these patients were also significantly higher than in those in whom Cr remained stable or decreased (adj. HR 1.9 [1.1‐3.4], *P* = .027).

Eighteen percent of HFrEF patients experienced deteriorating patterns of both urinary tubular biomarkers as well as Cr, while 31% of patients had at least one tubular biomarker rising without a change in Cr, and only 10% of patients had neither biomarker worsening during follow‐up (for details, see Figure 2C). Figure 2C displays that when tubular damage markers were stable or improving, Cr incline did not affect endpoint‐free rates. However, if either NAG or KIM‐1 slope increased, endpoint‐free rates decreased. Finally, the lowest endpoint‐free rates were in patients who had increasing slopes of all three renal biomarkers (*P* for trend <.001).

## 4 DISCUSSION

To our best knowledge, this study is the first to identify clinical determinants of progressive renal tubular damage in CHF. Of note, these determinants differ from those found for WRF, which strengthens the recommendation that glomerular and tubular compartment should be jointly assessed. This study also displays that patients in whom both renal compartments deteriorate during outpatient follow‐up have the lowest endpoint‐free survival.

Renal function may act as a “barometer” of the severity of CHF.^27^, ^28^ However, because of the multifactorial nature of cardiorenal interactions, merely assessing the glomerular function may be suboptimal for decision‐making. Our study confirms this and provides additional evidence for the notion that each aspect of the kidney (glomerular and tubular) provides incremental prognostic information, and together they may further identify higher‐risk CHF individuals. These kidney‐specific signals may therefore help physicians to better and timely target medical therapy before the future event occurs.

Based on our findings, we could speculate that “renoprotective” treatment targeted at the tubules may be even more effective than treatment simply aiming at improving only eGFR or Cr values. To this end, we have previously found that higher ACE‐inhibitor/ARBs doses during follow‐up were associated with less renal tubular damage together with less cardiac impairment (as assessed by NT‐proBNP and troponin levels).^29^ However, interventional studies on these tubular damage markers are needed to provide definite answers in this matter.

Our findings suggest that patients who have reduced eGFR already at baseline are more susceptible to tubular injury during follow‐up than those with higher baseline eGFR (ie, greater renal functional capacity). This phenomenon may potentially be attributed to the “work‐overload” in residual nephrons to compensate renal function in patients who had fewer functioning nephrons available.^30^ Compensatory hyperfiltration in the rest of nephrons may eventually exceed tubular adaption to hypoperfusion leading to tubulointerstitial hypoxic damage.^31^, ^32^ These intrinsic adaptations of tubules and peritubular capillaries to renal injury have been recognized as important factors for glomerulotubular balance to parallel glomerular filtration rate of a nephron.^33^

Moreover, these patients more frequently had diabetes which, on its part, may also have contributed to tubular injury. Similarly, other clinical determinants such as aging kidneys and severity of HF (higher cardiac markers and NYHA class, lower LV ejection fraction, and CRT) suggest that factors that are related to more severe HF also cause tubule‐specific renal injury. Importantly, renal tubular biomarkers entailed unfavorable outcomes even in patients with apparently stable glomerular function during follow‐up. Thus, the rise in urinary tubular biomarkers may indicate subclinical renal impairment even before renal function itself declines. Finally, our findings suggest that simultaneous assessment of NAG and KIM‐1 translates into better risk stratification in terms of survival rates than assessment of either marker alone.

Higher doses of loop diuretics and lower MRA doses were identified in patients with WRF and are supported by previous studies.^1^, ^34^ WRF was found to be associated with higher baseline eGFR which is also supported by several previous studies.^35^, ^36^, ^37^ However, this finding is inconsistent with the general opinion that WRF (defined as delta Cr >0.03 mg/dL) occurs more frequently in CHF patients that have impaired GFR already at baseline.^38^ One explanation for this discrepancy may be that closer monitoring of patients who already had impaired GFR could have also increased the likelihood of finding WRF in these patients,^34^ and particularly if sampling was not fixed but left at the discretion of the treating physician.^39^ As for our study, the observations were made using more than twice as many repeated measurements as in each of the previous studies, samples were collected prospectively at fixed time intervals defined by the study protocol. This further strengthens our suggestion that WRF should not be disregarded in CHF patients with relatively intact renal function.

Several limitations merit consideration. First, this study lacked direct GFR measurement. Second, we cannot comment on the effects of glomerular permeability on clinical outcomes since proteinuria was not measured. Third, causal inference is limited by the observational nature of our study. Although trials on this subject are still lacking, the repeated‐measures design of this study allows for stronger claims of true associations than previous studies do.

## 5 CONCLUSIONS

Slopes of tubular damage and WRF biomarkers had different clinical determinants. Both predicted clinical outcome, but this association was stronger for tubular injury. Prognostic effects of both appeared independent and additive. These findings are of particular interest since in current clinical practice the degree of tubular injury usually remains undetermined.

## CONFLICT OF INTEREST

The authors declare no potential conflict of interests.

## Supporting information


**Figure S1** Inclusion and exclusion criteria.

**Figure S2** Graphical depiction of the slope of biomarker trajectory during follow‐up.
The X‐axis depicts follow‐up time in months starting from baseline. The Y‐axis depicts biomarker level, and the black dots represent actually measured biomarker values during follow‐up. For each patient, the patient‐specific biomarker trajectory is constructed using joint models, which combine linear mixed‐effects models for the longitudinal biomarker trajectory with relative risk models for the time to event process, thus accounting for different follow‐up durations. In this figure, this estimated trajectory is displayed as a solid red line. The joint model inherently accounts for the biological variation that the biomarker may exhibit, but also for settings where extreme values are observed but are not particularly helpful clinically.^1^ The slope of the biomarker's trajectory (illustrated by the gray triangle) is then calculated as the first derivative of the function, and indicates whether and by how much the levels are increasing or decreasing, or whether they remain stable over time. Both blood (for creatinine) and urine (for tubular markers: NAG and KIM‐1) samples were collected simultaneously at fixed 3‐month intervals.

**Table S1** Baseline characteristics of HFrEF patients in the Bio‐SHiFT cohort.

**Table S2** Slopes of renal biomarkers according to baseline eGFR and study endpoints.

**Table S3** Total daily dose equivalents and conversion factors for ACE‐inhibitors/ARBs, β blockers, MRAs and loop diuretics/thiazides.
