# Supplementary figures and images for: Renal tubular damage and worsening renal function in chronic heart failure: Clinical determinants and relation to prognosis (Bio‐SHiFT study)
Source: Clin Cardiol. 2020 Apr 16;43(6):630–8. doi: 10.1002/clc.23359 (PMC7298997; doi:10.1002/clc.23359)

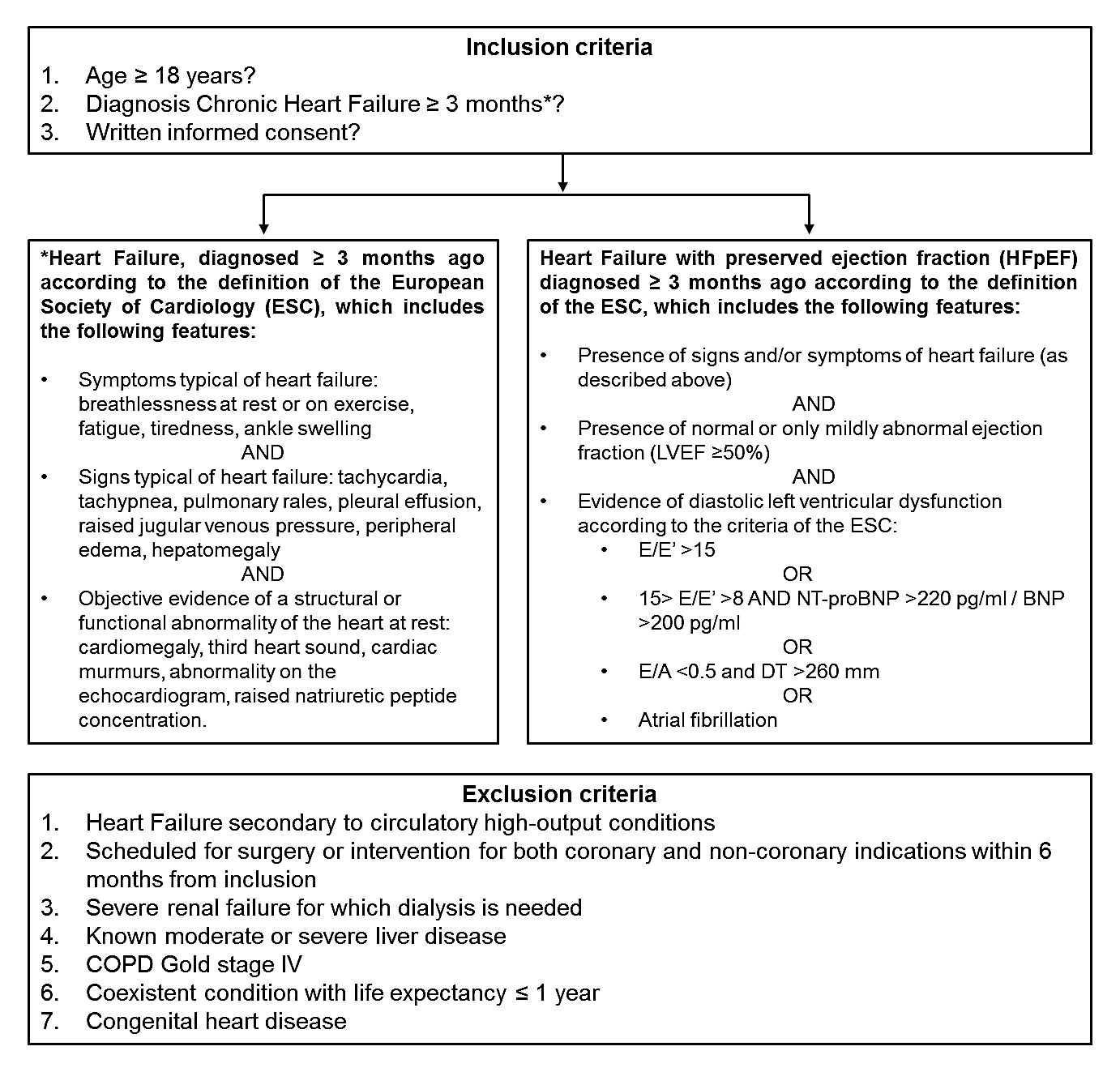
 **Figure S1. Inclusion and exclusion criteria.**

Supplement: Supplementary file 1 — Figure S1 Inclusion and exclusion criteria. [file CLC-43-630-s001.docx]
