# Supplementary material for: Renal tubular damage and worsening renal function in chronic heart failure: Clinical determinants and relation to prognosis (Bio‐SHiFT study)
Source: Clin Cardiol. 2020 Apr 16;43(6):630–8. doi: 10.1002/clc.23359 (PMC7298997; doi:10.1002/clc.23359)
Supplement: Supplementary file 2 — Figure S2 Graphical depiction of the slope of biomarker trajectory during follow‐up. The X‐axis depicts follow‐up time in months starting from baseline. The Y‐axis depicts biomarker level, and the black dots represent actually measured biomarker values during follow‐up. For each patient, the patient‐specific biomarker trajectory is constructed using joint models, which combine linear mixed‐effects models for the longitudinal biomarker trajectory with relative risk models for the time to event process, thus accounting for different follow‐up durations. In this figure, this estimated trajectory is displayed as a solid red line. The joint model inherently accounts for the biological variation that the biomarker may exhibit, but also for settings where extreme values are observed but are not particularly helpful clinically.1 The slope of the biomarker's trajectory (illustrated by the gray triangle) is then calculated as the first derivative of the function, and indicates whether and by how much the levels are increasing or decreasing, or whether they remain stable over time. Both blood (for creatinine) and urine (for tubular markers: NAG and KIM‐1) samples were collected simultaneously at fixed 3‐month intervals. [file CLC-43-630-s002.docx]

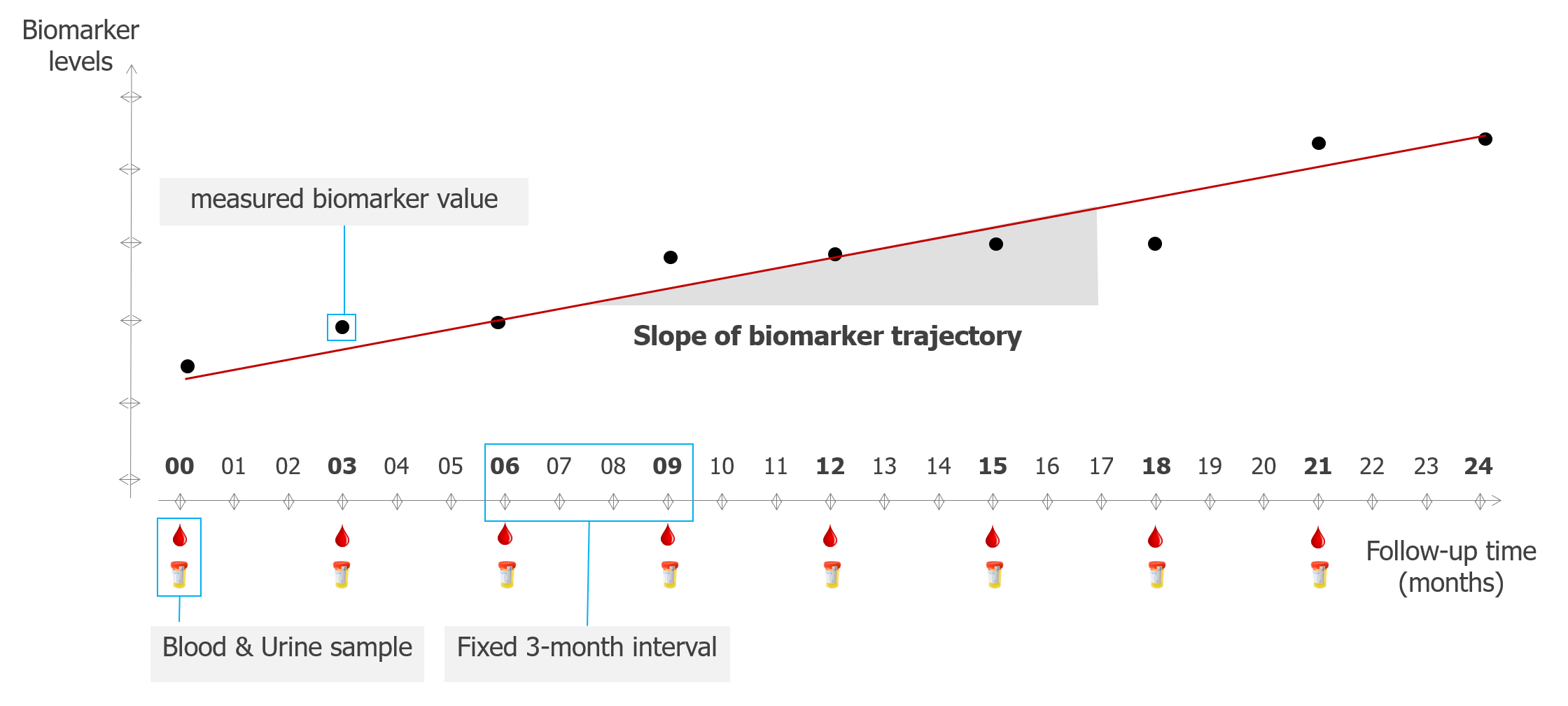
**Figure S2. Graphical depiction of the slope of biomarker trajectory during follow-up.**

The X-axis depicts follow-up time in months starting from baseline. The Y-axis depicts biomarker level, and the black dots represent actually measured biomarker values during follow-up. For each patient, the patient-specific biomarker trajectory is constructed using joint models, which combine linear mixed-effects models for the longitudinal biomarker trajectory with relative risk models for the time to event process, thus accounting for different follow-up durations. In this figure this estimated trajectory is displayed as a solid red line. The joint model inherently accounts for the biological variation that the biomarker may exhibit, but also for settings where extreme values are observed but are not particularly helpful clinically.^1^ The slope of the biomarker’s trajectory (illustrated by the gray triangle) is then calculated as the first derivative of the function, and indicates whether and by how much the levels are increasing or decreasing, or whether they remain stable over time. Both blood (for creatinine) and urine (for tubular markers: NAG and KIM-1) samples were collected simultaneously at fixed 3-month intervals.

Reference:

1. Brankovic M, Kardys I, Hoorn EJ, Baart S, Boersma E, Rizopoulos D. Personalized dynamic risk assessment in nephrology is a next step in prognostic research. *Kidney international.* 2018;94(1):214-217.
