# Supplementary material for: Renal tubular damage and worsening renal function in chronic heart failure: Clinical determinants and relation to prognosis (Bio‐SHiFT study)
Source: Clin Cardiol. 2020 Apr 16;43(6):630–8. doi: 10.1002/clc.23359 (PMC7298997; doi:10.1002/clc.23359)
Supplement: Supplementary file 3 — Table S1 Baseline characteristics of HFrEF patients in the Bio‐SHiFT cohort. [file CLC-43-630-s003.docx]

**Table S1. Baseline characteristics of HFrEF patients in the Bio-SHiFT cohort.**

|  | **Total**  **(n=250)** | **Composite endpoint reached** | |
| --- | --- | --- | --- |
|  |  | **Yes**  **(n=66)** | **No**  **(n=184)** |
| **Clinical features** |  |  |  |
| Age years | 66 ± 13 | 69 ± 13 | 65 ± 12 |
| Men, n (%) | 184 (74) | 52 (79) | 132 (72) |
| Ischemic etiology | 116 (46) | 36 (54) | 80 (43) |
| BMI kg/m^2^ | 27.4 ± 4.7 | 27.3 ± 4.7 | 27.5 ± 4.7 |
| Heart rate b.p.m. | 67 ± 11 | 68 ± 13 | 66 ± 11 |
| SBP mmHg | 122 ± 21 | 116 ± 18 | 123 ± 21 |
| DBP mmHg | 72 ± 11 | 70 ± 10 | 73 ± 11 |
| Congestion | 157 (63) | 53 (80) | 104 (57) |
| NYHA III/IV | 62 (25) | 29 (44) | 33 (18) |
| CRT | 78 (31) | 19 (29) | 59 (32) |
| **Echocardiographic features** | |  |  |
| LVEF, % | 30 (23 to 37) | 25 (19 to 34) | 30 (23 to 38) |
| DiasLVD, mm | 64 (57 to 71) | 68 (56 to 76) | 63 (57 to 70) |
| SysLVD, mm | 50 (42 to 59) | 55 (42 to 64) | 49 (42 to 58) |
| E/A ratio | 0.8 (0.6 to 1.4) | 1.4 (0.8 to 3.0) | 0.8 (0.6 to 1.2) |
| E/E' ratio | 10.4 (6.6 to 16.5) | 16.0 (9.0 to 21.7) | 9.8 (6.4 to 13.4) |
| **Medical history** |  |  |  |
| Prior MI | 95 (38) | 32 (48) | 63 (34) |
| Atrial fibrillation | 97 (39) | 33 (50) | 64 (35) |
| Diabetes | 77 (31) | 29 (44) | 48 (26) |
| Hypertension | 113 (45) | 34 (51) | 79 (43) |
| COPD | 31 (12) | 12 (18) | 19 (10) |
| **Medication** prevalence (%) /average total daily dose (mg) | | |  |
| Beta-blocker | 90% / 44 mg | 86% / 41 mg | 91% / 45 mg |
| ACE-I/ARBs | 94% / 24 mg | 89% / 21 mg | 96% / 25 mg |
| Loop diuretics | 91% / 84 mg | 97% / 122 mg | 89% / 69 mg |
| MRAs | 70% / 23 mg | 76% / 23 mg | 67% / 23 mg |
| **Cardiac biomarkers** | |  |  |
| NT-proBNP ng/L | 1126 (380 o 2321) | 2516 (1492 to 4436) | 794 (246 to 1734) |
| cTnT ng/L | 17.7 (9.3–32.8) | 30.1 (19.7–48.6) | 13.8 (8.2–27) |
| **Renal glomerular indices (plasma)** | |  |  |
| Creatinine mg/dl | 1.18 (0.99–1.49) | 1.32 (1.02–1.51) | 1.17 (0.97–1.48) |
| eGFR_mL/min/1.73m2_ | 58 (42–77) | 53 (39–73) | 60 (44–78) |
| eGFR<60 | 130 (52) | 39 (59) | 91 (184) |
| **Renal tubular markers (urine)** | |  |  |
| NAG, U/gCr | 5.8 (3.7–9.1) | 7.9 (5.9–10.8) | 5.1 (3.2–8.0) |
| KIM1, ng/gCr | 489 (247–935) | 589 (260–1803) | 463 (236–901) |

ACE-I, angiotensin-converting enzyme inhibitors; ARB, angiotensin II receptor blockers; A, peak late filling velocity; BMI, Body mass index; COPD, chronic obstructive pulmonary disease CRP, C-reactive protein;; cTnT, cardiac troponin T; CVA, cerebrovascular accident; DBP, Diastolic blood pressure; DiasLVD, diastolic left ventricular diameter; E, peak early filling velocity; E', early diastolic mitral annular velocity; eGFR, estimated glomerular filtration rate; KIM1, kidney injury molecule-1; MI, myocardial infarction; MRA, mineralocorticoid receptor antagonist; NAG, N-acetyl-β-D-glucosaminidase; NYHA class, New York Heart Association class; SBP, Systolic blood pressure; SysLVD, systolic left ventricular diameter; TIA, transitory ischemic attack. For reasons of uniformity continuous variables are presented as medians (25^th^ to 75^th^ percentiles) and categorical variables are presented as n (%).
