# Supplementary material for: Renal tubular damage and worsening renal function in chronic heart failure: Clinical determinants and relation to prognosis (Bio‐SHiFT study)
Source: Clin Cardiol. 2020 Apr 16;43(6):630–8. doi: 10.1002/clc.23359 (PMC7298997; doi:10.1002/clc.23359)
Supplement: Supplementary file 4 — Table S2 Slopes of renal biomarkers according to baseline eGFR and study endpoints. [file CLC-43-630-s004.docx]

**Table S2. Slopes of renal biomarkers according to baseline eGFR and study endpoints.**

| **Biomarker slopes** | | **adj. β (95% CI)** | **p-value** |
| --- | --- | --- | --- |
| **NAG** (urine) | |  |  |
|  | Baseline eGFR (per 10 mL/min/1.73m^2^ increase) | -0.02 (-0.04 to -0.01) | 0.012 |
|  | Study endpoint (yes) | 0.28 (0.21 to 0.36) | <0.001 |
|  | Interaction (eGFR x study endpoint) | ** | 0.90 |
| **KIM1** (urine) | |  |  |
|  | Baseline eGFR (per 10 mL/min/1.73m^2^ increase) | -0.02 (-0.03 to -0.01) | 0.020 |
|  | Study endpoint (yes) | 0.26 (0.18 to 0.34) | <0.001 |
|  | Interaction (eGFR x study endpoint) | ** | 0.53 |
| **Creatinine** (plasma) | |  |  |
|  | Baseline eGFR (per 10 mL/min/1.73m^2^ increase) | 0.02 (0.01 to 0.04) | 0.001 |
|  | Study endpoint (yes) | 0.20 (0.13 to 0.26) | <0.001 |
|  | Interaction (eGFR x study endpoint) | ** | 0.24 |

** Coefficient not presented since interaction was not significant; CI, confidence interval
