# Supplementary material for: Renal tubular damage and worsening renal function in chronic heart failure: Clinical determinants and relation to prognosis (Bio‐SHiFT study)
Source: Clin Cardiol. 2020 Apr 16;43(6):630–8. doi: 10.1002/clc.23359 (PMC7298997; doi:10.1002/clc.23359)
Supplement: Supplementary file 5 — Table S3 Total daily dose equivalents and conversion factors for ACE‐inhibitors/ARBs, β blockers, MRAs and loop diuretics/thiazides. [file CLC-43-630-s005.docx]

**Table S3. Total daily dose equivalents and conversion factors for ACE-inhibitors/ARBs, β blockers, MRAs and loop diuretics/thiazides.**

| **Drug Category** | **Maximal Dose**  **(Target Dose)** | **Equivalency Conversion** |
| --- | --- | --- |
| **ACE-inhibitors** | Total Daily Dose (mg) | Enalapril Dose Conversion Factor |
| Enalapril | 40 | x 1 |
| Lisinopril | 40 | x 1 |
| Captopril | 150 | / 3.75 |
| Quinapril | 40 | x 1 |
| Ramipril | 10 | x 4 |
| Fosinopril | 40 | x 1 |
| Perindopril | 16 | x 2.5 |
| Trandolapril | 4 | x 10 |
| **ARB** | Total Daily Dose (mg) | Enalapril Dose Conversion Factor |
| Candesartan | 32 | x 1.25 |
| Losartan | 50 | / 1.25 |
| Valsartan | 320 | / 8 |
| Irbesartan | 150 | /3.75 |
| **Β-blockers** | Total Daily Dose (mg) | Carvedilol Dose Conversion Factor |
| Carvedilol | 50 | x 1 |
| Bisprolol | 10 | x 5 |
| Metoprolol tartrate | 100 | / 2 |
| Atenolol | 50 | x 1 |
| Celiprolol | 200 | / 4 |
| Labetalol | 100 | / 2 |
| Nebivolol | 10 | x 5 |
| **Aldosterone Antagonists** | Total Daily Dose (mg) | Spironolactone Dose Conversion Factor |
| Spironolactone | 25 | x 1 |
| Eplerenone | 50 | / 2 |
| **Loop Diuretic/thiazides** | Total Daily Dose (mg) | Furosemide Dose Conversion Factor |
| Furosemide | 40 | x 1 |
| Bumetanide | 1 | x 40 |
| Torsemide | 20 | x 2 |
| Hydrochlorothiazide | 12.5 | *3.2 |
| Chlorothiazide | 36 | *1.44 |
